# Clinical outcomes of umbilical hernia in patients with liver cirrhosis: A retrospective comparative analysis of management strategies

**DOI:** 10.5339/qmj.2026.61

**Published:** 2026-09-08

**Authors:** Esraa Arabi, Abdalrhman Qazli, Ali Alamri, Yazed Albogami, Fatmah Alsohaibani, Ibrahim Albabtain, Zeyad Alyousef

**Affiliations:** 1 General Surgery Department, King Abdulaziz Medical City, Riyadh, Saudi Arabia; 2 General Surgery Department, Prince Mohammed Bin Abdulaziz Hospital, Riyadh, Saudi Arabia; 3 Thoracic Surgery Department, King Saud Medical City, Riyadh, Saudi Arabia; 4 King Saud bin Abdulaziz University for Health Sciences, Riyadh, Saudi Arabia

**Keywords:** Umbilicus, hernia, liver cirrhosis, herniorrhaphy, general surgery

## Abstract

**Background:**

Managing umbilical hernia in cirrhotic patients is complex. No prior national studies have examined the outcomes associated with treatment approaches for this demographic.

**Objectives:**

To evaluate the results of cirrhotic patients with umbilical hernia treated conservatively compared to those who underwent elective surgical hernia repair.

**Methodology:**

This is a retrospective cohort study conducted over 5 years period from January 2017 to December 2022 at King Abdulaziz Medical City and King Abdullah Specialized Children’s Hospital in Riyadh, Saudi Arabia. A comparison was conducted between those who underwent elective umbilical hernia repair and those treated conservatively. A chi-square test was used for categorical variables, while t-test and analysis of variance were used for numerical and categorical variables.

**Results:**

The study included 70 patients, out of which 54 patients initially planned for conservative treatment, and 21 eventually underwent emergent umbilical hernia repair. In the elective surgery group, 11 (68.8%) had a Child A score, whereas the emergency group had 9 patients (42.9%) with a Child B score and 10 patients (47.6%) with a Child C score (*p* = 0.001). Post-operatively, 12 patients (57.1%) required intensive care unit (ICU) admissions in the emergency group compared to only 2 patients (12.5%) in the elective group (*p* = 0.006).

**Conclusion:**

Expectant management of cirrhotic patients with umbilical hernia and ascites often results in a higher complication rate, frequently leading to emergency surgery, which is associated with significant morbidity. In contrast, elective hernia repair typically results in fewer complications and is therefore recommended.

## 1. INTRODUCTION

Ventral abdominal hernias are common among cirrhotic patients, with an incidence rate ranging from 20% to 40%, depending on the degree of liver compensation.^1–3^ The most common underlying cause of umbilical hernia in these patients is elevated intra-abdominal pressure, which forces the peritoneum to fold through the umbilicus, causing eversion.^4^ Portal hypertension also contributes to umbilical hernia development through recanalization of the obliterated umbilical vein or round ligament, thereby increasing the defect in the umbilical fascia and leading to herniation.^4,5^

Previous studies have reported a high mortality rate, ranging from 16% to 31%, for abdominal hernia repair in patients with cirrhosis.^6^ This is primarily because poorly controlled ascites is associated with post-operative complications, including surgical site infection, peritonitis, abdominal evisceration, and ascitic leak.^7^ These complications, along with a recurrence rate that can reach 60%, led to the adoption of a “wait and see” approach.^6^ However, patients managed expectantly also faced high rates of hernia incarceration, evisceration, ascitic leakage, and peritonitis.^5^ Among cirrhotic patients with abdominal hernias treated conservatively, 22.7% experienced complications necessitating emergency surgery.^6^ In cases with a Child-Pugh score of C, emergency hernia repair resulted in mortality rates as high as 57%.^6^ A study of 552 cirrhotic patients showed a 30-day mortality rate of 1.2% for those repaired electively, compared to 12.2% for those undergoing emergency procedures.^7^ Emergency surgery was performed in 22.7% of patients managed conservatively, with these individuals exhibiting higher mortality and complication rates compared to those who underwent elective surgery.^7^ Elective hernia repair allows for optimal management of a patient’s ascites through medical treatment, paracentesis, or, in refractory cases, the placement of a transjugular intrahepatic portosystemic shunt.^6^ Elective surgery for hernia repair can be considered in patients with Child-Pugh class A or a MELD score below 10. However, patients classified as Child-Pugh B/C or with a Model for End-Stage Liver Disease (MELD) score greater than 10 should receive appropriate perioperative management for ascites.^7^ A high risk of morbidity and mortality exists for those with a MELD score above 14 or Child-Pugh class C; therefore, hernia repair concurrent with liver transplantation is recommended.^7^

In summary, managing umbilical hernia surgery in cirrhotic patients is both complex and influenced by multiple factors. No prior national studies have examined the outcomes associated with treatment approaches for this demographic, as searched in PubMed, Scopus, Web of Science, Cochrane, and Google Scholar. Our study aims to evaluate the results of cirrhotic patients with umbilical hernias treated conservatively compared to those who underwent elective hernia repair at two tertiary centers in Riyadh, Saudi Arabia.

## 2. METHODOLOGY

This retrospective cohort study aimed to evaluate outcomes in cirrhotic patients with umbilical hernia, comparing those treated conservatively to those who underwent elective surgical repair. The study involved the Departments of General Surgery, Hepatology, and Hepatobiliary at King Abdulaziz Medical City and King Abdullah Specialized Children’s Hospital in Riyadh, Saudi Arabia. All patients were included, and the sample size was 70 patients.

### 2.1. Inclusion criteria

- Patients diagnosed with liver cirrhosis and umbilical hernia who visited our hospital over a five-year period from January 2017 to December 2022.
- Patients diagnosed with cirrhosis and umbilical hernia aged over 18.

### 2.2. Exclusion criteria

- Patients identified as support care or do not resuscitate.
- Patients who lost to follow-up.
- Individuals with a previous surgical abdominal history.
- Those who received umbilical hernia repair at the time of liver transplant.

Data were extracted using the Best Care electronic system and entered into a unified Excel data collection sheet. The collected data comprised age, gender, Child score, MELD score, MELD-Na score, and treatment of ascites (e.g., medical treatment, therapeutic paracentesis, transjugular intrahepatic portosystemic shunt procedure). It also included the management plan for umbilical hernia (conservative or elective surgical repair), the need for emergency surgery, surgical details (e.g., primary or mesh, laparoscopy or open, type of mesh, mesh placement method, type of sutures, and size of hernia), post-operative course (e.g., length of hospital stay, need for intensive care unit (ICU) admission, presence of post-operative liver decompensation), post-operative complications (e.g., surgical site infection, surgical bed collection, recurrence rate), and 30-day mortality.

### 2.3. Data analysis technique

The analysis was conducted in three groups. The first group compared patients who underwent elective umbilical hernia repair to those treated conservatively. The second group compared patients who had elective repairs to those who required emergency intervention. The third group examined hernia repair outcomes in relation to the three Child scores. Data analysis was conducted using the Statistical Package for the Social Sciences (SPSS), version 23. Categorical variables were displayed using frequencies and percentages, while numerical variables were presented with means and standard deviations. To examine associations between categorical variables, we employed the chi-square test. Associations between numerical and categorical variables were assessed using the independent t-test and analysis of variance (ANOVA). The level of significance was set at 0.05.

This research was approved by the King Abdullah International Medical Research Center with Institutional Review Board (IRB) approval number IRB/3213/23 on 26 December 2023. No consent was required due to the retrospective nature of the study.

## 3. RESULTS

The study included a total of 70 participants. Table 1 compares patients who had their umbilical hernia repaired electively with those treated conservatively. Of the elective surgery group, 11 patients (68.8%) had a Child A score. Meanwhile, 26 (48.1%) of the conservatively managed patients had a Child B score, showing a *p*-value of < 0.001. The conservative group exhibited higher MELD scores (19.11 ± 8.37) and MELD-Na scores (20.15 ± 8.54), with *p*-values of 0.02 and 0.009, respectively. In the conservative group, 49 patients (90.7%) received medical treatment for ascites compared to 11 patients (68.8%) in the elective surgery group (*p* = 0.027). Similarly, therapeutic paracentesis for ascites in the conservative group was done for 40 patients (74.1%) in contrast to 5 patients (31.3%) in the elective group (*p* = 0.02).

Table 2 compares patients who underwent elective umbilical hernia repair with those treated on an emergency basis. In the elective surgery group, 11 patients (68.8%) had a Child A score, whereas the emergency group had 9 patients (42.9%) with a Child B score and 10 patients (47.6%) with a Child C score (*p* = 0.001). The emergency group also exhibited higher MELD (19.05 ± 8.58) and MELD-NA scores (21.14 ± 8.92), with p-values of 0.049 and 0.013, respectively. Therapeutic paracentesis for ascites was done in 16 patients (76.2%) in the emergency surgery group and 5 patients (31.3%) in the elective group (*p* = 0.006). Post-operatively, 12 patients (57.1%) required ICU admissions in the emergency group compared to only 2 patients (12.5%) in the elective group (*p* = 0.006). There was no significant difference between the groups regarding age, gender, medical treatment for ascites, transjugular intrahepatic portosystemic shunting (TIPS) for ascites, type of repair (primary or mesh), surgical approach (open or laparoscopic), type of suture, hernia size, length of post-operative stay, post-operative decompensation, surgical site infection, surgical bed collection, recurrence after one year, and 30-day post-operative mortality.

Table 3 presents a comparison of patients who underwent hernia repair across the three Child scores. All 12 Child C patients received therapeutic paracentesis, whereas 7 (58.3%) of Child B patients did so (*p* < 0.001). Child B patients exhibited the highest rate of primary repair in 7 patients (58.3%), while mesh repair was done in 12 (92.3%) of Child A patients, with a *p*-value of 0.019. A statistically significant difference among the child scores was observed in the following: the type of surgery (*p* = 0.027), the length of hospital stays (*p* < 0.001), the ICU admission rate post-operatively (*p* < 0.001), and the rate of post-operative decompensation (*p* < 0.001). Age, gender, medical treatment for ascites, TIPS for ascites, type of suture, size of hernia, surgical site infection, surgical bed collections, recurrence rate after one year of follow-up, and 30-day post-operative mortality were not significantly associated with the Child score.

## 4. DISCUSSION

The management of cirrhotic patients with umbilical hernias has historically been controversial.^8^ Initially, expectant management was the preferred approach, as surgery in cirrhotic patients was deemed high-risk. Consequently, many surgeons opted to delay umbilical hernia repair until bowel-related complications (e.g., incarceration, strangulation, rupture, ulceration, or ascitic fluid leakage) were present.^9^

### 4.1. Elective surgery versus expectant management

Marsman et al.^10^ compared elective surgical repair (*n* = 17) with expectant treatment (*n* = 13) in cirrhotic patients with umbilical hernia and ascites. Their findings indicated that expectant treatment led to increased morbidity and mortality. Hospital admission due to hernia incarceration occurred in ten of the 13 patients (77%), with six requiring emergency herniorrhaphy. Furthermore, two patients (15%) receiving expectant treatment died from hernia complications. Conversely, among those who underwent elective herniorrhaphy, there were no complications or hernia recurrences in 12 of the 17 patients (71%).^10^ In a study by de Goede et al.^11^ 16 patients were randomly assigned to elective repair and 18 to conservative treatment. After 24 months, complications related to the umbilical hernia or its repair occurred in eight patients (50%) undergoing elective repair and in 14 patients (77.8%) undergoing conservative treatment. Overall, several studies have demonstrated superior outcomes with elective umbilical herniorrhaphy, recommending it to prevent complications associated with conservative management.^8,9,12^ In our study, 54 patients underwent expectant management, 26 (48.1%) of which were child B and 20 (37%) were child C. Emergency surgery was required for 21 patients due to hernia complications.

### 4.2. Elective surgery versus emergency surgery

Over the past decade, numerous studies have questioned the traditional approach to umbilical hernia repair due to high morbidity and mortality rates among cirrhotic patients undergoing emergency procedures. As a result, elective surgical repair has been advocated, with outcomes largely dependent on ascites control and liver function grading. Saleh et al. examined 30-day mortality among 688 cirrhotic patients with ascites who underwent either elective or emergency umbilical hernia surgery. In this study, 300 patients (44%) underwent emergency surgery, with 27 deaths (9%) occurring compared to 18 deaths out of 388 patients (4.6%) who had elective surgery (*p* = 0.02). The study concluded that emergency surgery, albumin levels below 30 g/L, INR ≥ 1.6, thrombocytopenia, MELD score ≥ 15, and renal insufficiency were independent predictive factors for short-term mortality (≤ 30 days).^13^ The mean length of hospital stay increased with higher Child-Pugh scores and was further prolonged in emergency settings.^14,15^ Readmission rates also differed significantly; for instance, in emergency situations, rates were 13.51% for compensated cirrhosis and 34.55% for decompensated cirrhosis, compared to 11.39% in patients without cirrhosis.^15^ For elective cases, readmission rates were 7.23% for compensated cirrhosis and 16.42% for decompensated cirrhosis, compared to 4.87% in patients without cirrhosis.^15^ Another study reported readmission rates of 27% for emergency settings and 14% for elective settings.^16^ In our study, the emergency surgical group exhibited a higher rate of ICU admission post-operatively in comparison to the elective group. However, no statistically significant difference was observed in the length of hospital stay, post-operative decompensation, surgical site collection, infection, or mortality (Table 2).

### 4.3. Peri-operative optimization and hernia recurrence

Hernia recurrence was observed in 45% of patients with uncontrolled ascites, compared to 4% in those with controlled ascites. This underscores the importance of optimizing patients with liver cirrhosis before elective umbilical hernia repair to minimize post-operative complications and reduce recurrence risk.^5,17^ Recommended strategies include dietary modifications, such as low salt intake and free water restriction, alongside pharmacologic management with diuretics. In cases of refractory ascites, more invasive interventions, like TIPS or the placement of a temporary peritoneal dialysis catheter, may be considered during hernia repair to mitigate perioperative risks.^17^ Several risk factors have been identified as predictors of post-operative mortality, including age over 65, a MELD score greater than 15, albumin levels less than 3.0 g/dL, and preoperative sepsis.^18^ The European Hernia Society’s latest guidelines, published in 2019, recommend that patients with cirrhosis may undergo elective surgery if their MELD score is below 15 and/or if they have Child-Pugh A cirrhosis, with or without preoperative liver function optimization.^19^ In our study, patients with higher Child scores experienced a statistically significant longer duration of hospital stay and ICU admission rate, as well as a higher rate of decompensation in both the elective and emergency groups (Table 3). Nevertheless, there was no statistically significant difference in hernia recurrence between the groups over one year (Table 2).

### 4.4. Surgical technique

Laparoscopic umbilical hernia repair offers notable advantages over traditional open repair, especially for patients with liver cirrhosis. This approach provides a tension-free repair, minimizing ascites leakage and trauma to large collateral veins. As a result, there is reduced blood loss, less post-operative pain, better cosmetic outcomes, faster recovery, and a lower risk of hernia recurrence. These benefits make it a preferred option for this patient group.^20–22^ Elective mesh umbilical herniorrhaphy in cirrhotic patients with ascites has proven to be a safe intervention, with reports indicating a reduction in hernia recurrence.^10,12,23,24^

A study involving 80 cirrhotic patients undergoing umbilical repair demonstrated that polypropylene mesh significantly lowered the hernia recurrence rate compared to conventional fascial sutures (2.7% vs. 14.2%).^23^ Regarding surgical technique, the tension-free method with a mesh overlap of more than 2–3 cm is preferred. Sublay mesh repair is considered superior to other techniques due to its lower risk of recurrence and post-operative wound complications.^25–^ In our study, there was a statistically significant difference observed between the type of surgery and the type of hernia repair among the different Child scores (Table 2).

### 4.5. Limitations

This study is limited by its retrospective nature and the limited number of patients. Those with higher Child scores tend to be treated conservatively, limiting the assessment of the safety of elective surgical repair in advanced liver disease.

### 4.6. Clinical recommendations

Expectant management of cirrhotic patients with umbilical hernia and ascites often results in a higher complication rate, frequently leading to emergency surgery, which is associated with significant morbidity and mortality. In contrast, elective hernia repair typically results in fewer complications and is therefore recommended. Controlling ascites effectively is crucial for minimizing perioperative complications and preventing recurrence. For patients awaiting liver transplantation, umbilical herniorrhaphy should ideally be performed during the transplant procedure.^5^

## 5. CONCLUSION

To sum up, the emergency surgical group exhibited a higher rate of ICU admission post-operatively. However, no statistically significant differences were observed in the length of hospital stay, post-operative decompensation, surgical site collection or infection, recurrence within one year, or mortality. Patients with higher Child scores experienced a statistically significantly longer duration of hospital stay and ICU admission rates, as well as high rates of decompensation in both the elective and emergency groups.

## ACKNOWLEDGEMENT

The authors gratefully acknowledge the American manuscript editors for English language editing.

## AUTHORS’ CONTRIBUTIONS

EA: Writing – Original draft, Writing – review & editing, resources. AQ: Writing – Original draft, Writing – review & editing, resources. AA: Data curation, software & validation, investigation & analysis. YA: Data curation, investigation & analysis, Writing – Original draft. IA: Conceptualization, supervision. FA: Data curation, software & validation, investigation & analysis. ZA: Conceptualization, supervision.

## CONFLICT OF INTEREST

The authors have no conflicts of interest to declare.

## DISCLOSURE OF AI USE

The authors declare that no artificial intelligence (AI) tool was used in this study.

## DATA AVAILABILITY

The datasets generated and/or analyzed during the current study are available from the corresponding author on reasonable request.

## FUNDING

The authors received no financial support for the research, authorship, and/or publication of this article.

## ETHICAL APPROVAL

The institutional review board of King Abdullah International Medical Research Center, Riyadh, Saudi Arabia, approved this research with an approval number IRB/3213/23 and has complied with the Declaration of Helsinki.

## Figures and Tables

**Table 1.** Comparison between patients with conservative management and surgical management (*n* = 70).

Factor	Group		*p*-value
	Conservative surgical management (*n* = 54)	Elective surgical management (*n* = 16)	
Age (mean + standard deviation) (*n* = 70)	66.06 + 10.61	64.13 + 10.33	0.522
Gender (n, %) (*n* = 70)			0.695
Male	30 (55.6%)	8 (50%)	
Female	24 (44.4%)	8 (50%)	
Child score (*n*, %) (*n* = 70)			< 0.001*
Child A	8 (14.8%)	11 (68.8%)	
Child B	26 (48.1%)	3 (18.8%)	
Child C	20 (37%)	2 (12.5%)	
MELD score (*n* = 70)	19.11 + 8.37	13.63 + 7.15	0.02*
MELD NA score (*n* = 70)	20.15 + 8.54	13.63 + 8.21	0.009*
Medical treatment of ascites (*n*, %) (*n* = 70)			0.027*
Yes	49 (90.7%)	11 (68.8%)	
No	5 (9.3%)	5 (31.3%)	
Therapeutic paracentesis for ascites (*n*, %) (*n* = 70)			0.002*
Yes	40 (74.1%)	5 (31.3%)	
No	14 (25.9%)	11 (68.8%)	
TIPS for ascites (*n*, %) (*n* = 70)			0.435
Yes	2 (3.7%)	0 (0%)	
No	52 (96.3%)	16 (100%)	
Number who underwent emergency surgery			
Emergency	21	-	

*Indicates significant at level 0.05.

**Table 2.** Comparison between patients with elective and emergency surgical management (*n* = 37).

Factor	Group		*p*-value
	Elective surgical management	Emergency surgical management	
Age (mean + standard deviation) (*n* = 37)	64.13 + 10.33	67.71 + 9.64	0.284
Gender (*n*, %) (*n* = 37)			0.306
Male	8 (50%)	14 (66.7%)	
Female	8 (50%)	7 (33.3%)	
Child score (*n*, %) (*n* = 37)			0.001*
Child A	11 (68.8%)	2 (9.5%)	
Child B	3 (18.8%)	9 (42.9%)	
Child C	2 (12.5%)	10 (47.6%)	
MELD score (*n* = 37)	13.63 + 7.15	19.05 + 8.58	0.049*
MELD NA score (*n* = 37)	13.63 + 8.21	21.14 + 8.92	0.013*
Medical treatment of ascites (*n*, %) (*n* = 37)			0.214
Yes	11 (68.8%)	18 (85.7%)	
No	5 (31.3%)	3 (14.3%)	
Therapeutic paracentesis for ascites (*n*, %) (*n* = 37)			0.006*
Yes	5 (31.3%)	16 (76.2%)	
No	11 (68.8%)	5 (23.8%)	
TIPS for ascites (*n*, %) (*n* = 37)			0.204
Yes	0 (0%)	2 (9.5%)	
No	16 (100%)	19 (90.5%)	
**Details of the operative management**			
Surgical management (*n* = 37)			0.160
Primary	4 (25%)	10 (47.6%)	
Mesh	12 (75%)	11 (52.4%)	
Type of surgery (*n* = 37)			0.103
Open surgery	10 (62.5%)	18 (85.7%)	
Laprascopy	6 (37.5%)	3 (14.3%)	
Type of sutures (*n* = 37)			0.243
None	0 (0%)	1 (4.8%)	
PDS	3 (18.8%)	5 (23.8%)	
Polydioxanone	1 (6.3%)	6 (28.6%)	
Polyglactin	5 (31.5%)	2 (9.5%)	
Polypropylen	5 (31.5%)	4 (19%)	
Prolene	1 (6.3%)	3 (14.3%)	
Vicryl	1 (6.3%)	0 (0%)	
Size of hernia (*n* = 37)	3.01 + 2.22	2.96 + 1.90	0.941
**Post-operative course**			
Length of stay post operatively (*n* = 37)	7.75 + 8.20	10.33 + 6.86	0.304
ICU admission post operatively (*n*, %) (*n* = 37)			0.006*
Yes	2 (12.5%)	12 (57.1%)	
No	14 (87.5%)	9 (42.9%)	
Post operative decompensation (*n*, %) (*n* = 37)			0.086
Yes	1 (6.3%)	6 (28.6%)	
No	15 (93.8%)	15 (71.4%)	
Surgical site infection (*n*, %) (*n* = 37)			0.376
Yes	0 (0%)	1 (4.8%)	
No	16 (100%)	20 (95.2%)	
Surgical bed collections (*n*, %) (*n* = 37)			0.982
Yes	3 (18.8%)	4 (19%)	
No	13 (81.3%)	17 (81%)	
Recurrence rate after one year follow-up (*n*, %) (*n* = 37)			0.095
Yes	5 (31.3%)	2 (9.5%)	
No	11 (68.8%)	19 (90.5%)	
30 days mortality (*n*, %) (*n* = 37)			0.115
Yes	0 (0%)	3 (14.3%)	
No	16 (100%)	18 (85.7%)	

*Indicates significant at level 0.05.

**Table 3.** Comparison across different Child scores (*n* = 37).

Factor	Group			*p*-value
	Child A	Child B	Child C	
Age (mean + standard deviation) (*n* = 37)	64.92 +10.28	69.08 +10.06	64.58 + 9.76	0.478
Gender (*n*, %) (*n* = 37)				0.479
Male	6 (46.2%)	8 (66.7%)	8 (66.7%)	
Female	7 (53.8%)	4 (33.3%)	4 (33.3%)	
MELD score (*n* = 37)	11.77 + 6.13	17.42 + 7.65	21.33 + 8.69	0.011*
MELD NA score (*n* = 37)	12.23 + 7.29	19.83 + 8.85	22.08 + 9.26	0.016*
Medical treatment of ascites (*n*, %) (*n* = 37)				0.062
Yes	8 (61.5%)	9 (75%)	12 (100%)	
No	5 (38.5%)	3 (25%)	0 (0%)	
Therapeutic paracentesis for ascites (*n*, %) (*n* = 37)				< 0.001*
Yes	2 (15.4%)	7 (58.3%)	12 (100%)	
No	11 (84.6%)	5 (41.7%)	0 (0%)	
TIPS for ascites (*n*, %) (*n* = 37)				0.601
Yes	1 (7.7%)	0 (0%)	1 (8.3%)	
No	12 (92.3%)	12 (100%)	11 (91.7%)	
**Details of the operative management**				
Surgical management (*n* = 37)				0.019*
Primary	1 (7.7%)	7 (58.3%)	6 (50%)	
Mesh	12 (92.3%)	5 (41.7%)	6 (50%)	
Type of surgery (*n* = 37)				0.027*
Open surgery	7 (53.8%)	9 (75%)	12 (100%)	
Laprascopy	6 (46.2%)	3 (25%)	0 (0%)	
Type of sutures (*n* = 37)				0.543
None	0 (0%)	0 (0%)	1 (8.3%)	
PDS	2 (15.4%)	3 (25%)	3 (25%)	
Polydioxanone	1 (7.7%)	4 (33.3%)	2 (16.7%)	
Polyglactin	4 (30.8%)	1 (8.3%)	2 (16.7%)	
Polypropylen	3 (23.1%)	4 (33.3%)	2 (16.7%)	
Prolene	2 (15.4%)	0 (0%)	2 (16.7%)	
Vicryl	1 (7.7%)	0 (0%)	0 (0%)	
Size of hernia (*n* = 65)	3.15 + 2.4	2.23 + 0.79	3.56 +2.32	0.263
**Post-operative course**				
Length of stay post operatively (*n* = 41)	4.62 + 4.17	7 + 5.24	16.42 +7.04	< 0.001*
ICU admission post operatively (*n*, %) (*n* = 41)				< 0.001*
Yes	0 (0%)	4 (33.3%)	10 (83.3%)	
No	13 (100%)	8 (66.7%)	2 (16.7%)	
Post operative decompensation (*n*, %) (*n* = 41)				0.028*
Yes	0 (0%)	2 (16.7%)	5 (41.7%)	
No	13 (100%)	10 (83.3%)	7 (58.3%)	
Surgical site infection (*n*, %) (*n* = 41)				0.387
Yes	1 (1.1%)	0 (0%)	0 (0%)	
No	12 (92.3%)	12 (100%)	12 (100%)	
Surgical bed collections (*n*, %) (*n* = 41)				0.271
Yes	2 (15.4%)	1 (8.3%)	4 (33.3%)	
No	11 (84.6%)	11 (91.7%)	8 (66.7%)	
Recurrence rate after one year follow-up (*n*, %) (*n* = 41)				0.519
Yes	3 (23.1%)	3 (25%)	1 (8.3%)	
No	10 (76.9%)	9 (75%)	11 (91.7%)	
30 days mortality (*n*, %) (*n* = 42)				0.312
Yes	0 (0%)	1 (8.3%)	2 (16.7%)	
No	13 (100%)	11 (9.17%)	10 (83.3%)	

*Indicates significant at level 0.05.
